# Supplementary material for: Elevated Lactate Dehydrogenase Has Prognostic Relevance in Treatment-Naïve Patients Affected by Chronic Lymphocytic Leukemia with Trisomy 12
Source: Cancers (Basel). 2019 Jun 26;11(7):896. doi: 10.3390/cancers11070896 (PMC6678692; doi:10.3390/cancers11070896)
Supplement: Supplementary file 1 [file cancers-11-00896-s001.pdf]

Supplementary Materials

Table S1. Multivariate analysis in +12 CLL patients (Cox regression analysis).

	Univariate Analysis			Multivariate Analysis		
	**HR**	**95% CI**	**P**	**HR**	**95% CI**	**P**
PFS						
Age	1.05	0.84 to 1.31	0.674	-		-
Sex	1.12	0.88 to 1.41	0.358	-		-
Binet stage	2.33	1.95 to 2.79	<0.001	1.21	0.88 to 1.66	0.242
Rai stage	2.54	2.10 to 3.08	<0.001	2.24	1.59 to 3.16	<0.001
LDH	1.70	1.33 to 2.17	<0.001	1.65	1.23 to 2.22	0.001
ZAP70	1.19	0.91 to 1.55	0.196	-		-
CD38	1.14	0.90 to 1.45	0.276	-		-
β-2 micro	1.87	1.45 to 2.42	<0.001	1.41	1.05 to 1.91	0.023
IGHV	2.01	1.54 to 2.62	<0.001	1.74	1.29 to 2.36	<0.001
TFS						
Age	1.00	0.80 to 1.26	0.994	-		-
Sex	1.08	0.85 to 1.37	0.523	-		-
Binet stage	2.32	1.95 to 2.77	<0.001	1.30	0.94 to 1.79	0.115
Rai stage	1.62	1.47 to 1.78	<0.001	2.07	1.46 to 2.93	<0.001
LDH	1.85	1.45 to 2.37	<0.001	1.74	1.29 to 2.35	<0.001
ZAP70	1.37	1.05 to 1.80	0.022	-		-
CD38	1.12	0.88 to 1.43	0.374	-		-
β-2 micro	2.04	1.56 to 2.65	<0.001	1.47	1.08 to 1.99	0.013
IGHV	2.05	1.56 to 2.68	<0.001	1.87	1.38 to 2.54	<0.001
OS						
Age	2.26	1.56 to 3.26	<0.001	1.74	1.07 to 2.83	0.027
Sex	0.94	0.65 to 1.35	0.731	-		-
Binet stage	1.54	1.14 to 2.08	0.005	1.03	0.60 to 1.76	0.907
Rai stage	1.63	1.20 to 2.20	0.001	1.42	0.82 to 2.47	0.212
LDH	2.24	1.53 to 3.28	<0.001	1.94	1.19 to 3.16	0.008
ZAP70	1.65	1.07 to 2.54	0.024	-		-
CD38	1.50	1.01 to 2.23	0.043	-		-
β-2 micro	2.23	1.48 to 3.37	<0.001	2.07	1.24 to 3.44	0.005
IGHV	2.42	1.56 to 3.75	<0.001	1.70	1.04 to 2.77	0.033
CLL-specific svv						
Age	1.56	0.92 to 2.62	0.096	-		-
Sex	1.04	0.62 to 1.76	0.882	-		-
Binet stage	2.38	1.64 to 3.44	<0.001	1.44	0.68 to 3.08	0.342
Rai stage	2.56	1.68 to 3.91	<0.001	2.15	0.85 to 5.45	0.108
LDH	4.31	2.46 to 7.56	<0.001	3.78	1.73 to 8.26	0.001
ZAP70	1.94	1.04 to 3.64	0.039	-		-
CD38	1.22	0.71 to 2.10	0.477	-		-
β-2 micro	3.52	1.82 to 6.79	<0.001	3.09	1.31 to 7.27	0.010
IGHV	3.84	1.91 to 7.74	<0.001	1.79	0.81 to 3.99	0.151

HR: hazard ratio; CI: confidence interval; PFS: progression-free-survival; TFS: treatment-free-survival; LDH: Lactate DeHydrogenase; OS: overall survival; IGHV: immunoglobulin heavy-chain variable region gene; CLL: chronic lymphocytic leukemia Factors with $p < 0.01$ entered in Multivariate analysis (MV) analysis.

Table S2. Multivariate analysis in negative FISH CLL patients (Cox regression analysis).

	Univariate Analysis			Multivariate Analysis		
	**HR**	**95% CI**	**P**	**HR**	**95% CI**	**P**
PFS						
Age	1.23	1.00 to 1.51	0.045	-		-
Sex	1.28	1.04 to 1.58	0.021	-		-
Binet stage	2.80	2.43 to 3.23	<0.001	1.90	1.36 to 2.65	<0.001
Rai stage	2.89	2.47 to 3.38	<0.001	1.71	1.22 to 2.40	0.002
LDH	1.58	1.20 to 2.08	0.001	0.92	0.65 to 1.32	0.664
ZAP70	1.35	1.07 to 1.70	0.012	-		-
CD38	2.37	1.91 to 2.94	<0.001	1.59	1.19 to 2.12	0.002
β-2 micro	1.77	1.41 to 2.24	<0.001	1.57	1.20 to 2.05	0.001
IGHV	2.56	2.05 to 3.19	<0.001	1.53	1.15 to 2.03	0.003
TFS						
Age	1.31	1.05 to 1.63	0.015	-		-
Sex	1.28	1.02 to 1.61	0.031	-		-
Binet stage	3.18	2.75 to 3.67	<0.001	2.25	1.46 to 3.45	<0.001
Rai stage	3.32	2.82 to 3.91	<0.001	1.49	0.97 to 2.30	0.071
LDH	1.64	1.23 to 2.19	0.001	0.88	0.58 to 1.33	0.537
ZAP70	1.76	1.38 to 2.26	<0.001	1.06	0.76 to 1.49	0.724
CD38	2.53	2.01 to 3.19	<0.001	1.72	1.22 to 2.42	0.002
β-2 micro	2.05	1.60 to 2.61	<0.001	1.81	1.32 to 2.49	<0.001
IGHV	3.03	2.39 to 3.84	<0.001	2.02	1.41 to 2.91	<0.001
OS						
Age	4.62	3.20 to 6.67	<0.001	5.39	3.07 to 9.48	<0.001
Sex	1.21	0.86 to 1.70	0.276	-		-
Binet stage	2.36	1.92 to 2.89	<0.001	2.72	1.35 to 5.52	0.005
Rai stage	2.26	1.80 to 2.84	<0.001	0.79	0.40 to 1.58	0.510
LDH	1.19	0.75 to 1.87	0.464	-		-
ZAP70	2.07	1.45 to 2.96	<0.001	1.95	1.20 to 3.17	0.007
CD38	1.92	1.36 to 2.70	<0.001	1.47	0.89 to 2.42	0.133
β-2 micro	1.87	1.29 to 2.71	0.001	1.32	0.83 to 2.09	0.248
IGHV	2.86	1.99 to 4.12	<0.001	1.25	0.75 to 2.11	0.390
CLL-specific svv						
Age	3.43	2.05 to 5.73	<0.001	3.65	1.66 to 8.03	<0.001
Sex	1.09	0.67 to 1.78	0.728	-		-
Binet stage	2.94	2.21 to 3.90	<0.001	4.42	1.43 to 13.63	0.010
Rai stage	3.10	2.24 to 4.29	<0.001	0.73	0.24 to 2.24	0.586
LDH	1.59	0.87 to 2.93	0.135	-		-
ZAP70	2.60	1.52 to 4.48	0.001	2.94	1.38 to 6.27	0.005
CD38	2.86	1.76 to 4.65	<0.001	2.16	1.01 to 4.60	0.046
β-2 micro	2.66	1.56 to 4.53	<0.001	1.89	0.94 to 3.80	0.074
IGHV	3.78	2.16 to 6.59	<0.001	1.02	0.46 to 2.27	0.957

HR: hazard ratio; CI: confidence interval; PFS: progression-free-survival; TFS: treatment-free-survival; LDH: Lactate DeHydrogenase; OS: overall survival; IGHV: immunoglobulin heavy-chain variable region gene; CLL: chronic lymphocytic leukemia Factors with $p < 0.01$ entered in Multivariate analysis (MV) analysis.

Table S3. Baseline characteristics of the validation cohort.

		Patients with FISH +12 (250 Patients)
Median age (years)		60.5 (32–87)
Gender M/F (ratio)		147/103 (1.43)
Binet stage	A	n.a.
	B	
	C	
Rai stage	0	66 (26.4%)
	I–II	145 (58.0%)
	III–IV	39 (15.6%)
Palpable splenomegaly		40 (16.0%)
Palpable hepatomegaly		14 (5.6%)
Lymphadenopathies >5 cm		172 (68.8%)
White blood cells (mmc)		18.450 (11,800–39,200)
Lymphocytes peripheral blood (mmc)		12.025 (6.490–33.150)
Hemoglobin (g/dL)		13.6 (12.3–14.7)
Platelets (mmc)		199,500 (143,000–250,000)
LDH	Normal levels	145/249 (58.2%)
	Above the limit	104/249 (41.8%)
Lymphocytes bone marrow (%)		64 (47–78)
ZAP70 positive (≥20%)		98/221 (44.3%)
CD38 positive (≥30%)		109/198 (55.0%)
CD49d positive (≥30%)		n.a.
β-2-microglobulin	Normal levels	199/243 (81.9%)
	Above the limit	44/243 (18.1%)
IGHV mutational status	Mutated	84/187 (44.9%)
	Unmutated	103/187 (55.1%)

FISH: Fluorescence in situ hybridization; M/F: male/female; IGHV: immunoglobulin heavy-chain variable region gene. n.a. not available.

Table S4. Baseline characteristics of the validation cohort CLL patients with +12 divided in two subgroups according to LDH levels.

		Patients with High LDH Levels (104 Patients)	Patients with Normal LDH Levels (145 Patients)	p
Median age (years)		62.0 (33–80)	59.0 (32–87)	ns
Gender M/F (ratio)		61/43 (1.42)	85/60 (1.42)	ns
Binet stage	A	n.a.	n.a.	-
	B			
	C			
Rai stage	0	21 (20.2%)	44 (30.3%)	0.001
	I–II	59 (56.7%)	86 (59.3%)	
	III–IV	24 (23.1%)	15 (10.4%)	
Palpable splenomegaly		26 (25.0%)	14 (9.6%)	0.001
Palpable hepatomegaly		7 (6.7%)	7 (4.8%)	ns
Lymphadenopathies >5 cm		80 (76.9%)	92 (63.4%)	0.023
White blood cells (mmc)		37.700 (16,800-74,800)	13,900 (9300–21,500)	<0.001
Lymphocytes peripheral blood (mmc)		28.610 (10,790–62,100)	9.030 (4860–15,680)	<0.001
Hemoglobin (g/dL)		13.2 (11.9–14.4)	13.8 (12.8–14.9)	0.001
Platelets (mmc)		184,500 (125,500–239,500)	211,000 (146,000–258,000)	0.021

**Lymphocytes bone marrow (%)**		75 (62–84)	54 (38–71)	<0.001
**ZAP70 positive (≥20%)**		51/97 (52.6%)	47/123 (38.2%)	0.033
**CD38 positive (≥30%)**		47/84 (55.9%)	62/114 (54.4%)	ns
**CD49d positive (≥30%)**		n.a.	n.a.	-
**β-2microglobulin**	**Normal**<br>**High levels**	71/100 (71.0%)<br>29/100 (29.0%)	127/142 (89.4%)<br>15/142 (10.6%)	<0.001
***IGHV* mutational status**	**Mutated**<br>**Unmutated**	30/81 (37.0%)<br>51/81 (63.0%)	53/105 (50.5%)<br>52/105 (49.5%)	ns

LDH: Lactate DeHydrogenase; M/F: male/female; IGHV: immunoglobulin heavy-chain variable region gene. n.a. not available.

Table 5. Multivariate analysis in validation cohort patients (Cox regression analysis).

	**Univariate Analysis**			**Multivariate Analysis**		
	**HR**	**95% CI**	**P**	**HR**	**95% CI**	**P**
PFS						
Age	0.91	0.64 to 1.31	0.640	-		-
Sex	1.01	0.72 to 1.42	0.939	-		-
Rai stage	2.06	1.56 to 2.73	<0.001	1.08	0.68 to 1.82	0.814
LDH	2.12	1.52 to 2.97	<0.001	1.78	1.21 to 2.62	0.004
ZAP70	1.54	1.09 to 2.17	0.014	1.30	0.90 to 1.88	0.155
CD38	0.76	0.53 to 1.10	0.146	-		-
β-2 micro	2.07	1.40 to 3.05	<0.001	1.06	0.64 to 1.74	0.828
IGHV	1.22	0.84 to 1.76	0.297	-		-
TFS						
Age	0.91	0.64 to 1.31	0.640	-		-
Sex	1.01	0.72 to 1.42	0.939	-		-
Rai stage	2.06	1.56 to 2.73	<0.001	1.08	0.68 to 1.82	0.814
LDH	2.12	1.52 to 2.97	<0.001	1.78	1.21 to 2.62	0.004
ZAP70	1.54	1.09 to 2.17	0.014	1.30	0.90 to 1.88	0.155
CD38	0.76	0.53 to 1.10	0.146	-		-
β-2 micro	2.07	1.40 to 3.05	<0.001	1.06	0.64 to 1.74	0.828
IGHV	1.22	0.84 to 1.76	0.297	-		-
OS						
Age	4.00	2.03 to 7.87	<0.001	3.78	1.92 to 7.45	<0.001
Sex	1.19	0.61 to 2.32	0.601	-		-
Rai stage	1.41	0.80 to 2.48	0.231	-		-
LDH	2.29	1.18 to 4.47	0.015	2.01	1.01 to 3.99	0.047
ZAP70	1.42	0.70 to 2.89	0.337	-		-
CD38	0.94	0.46 to 1.91	0.854	-		-
β-2 micro	2.09	1.07 to 4.09	0.031	1.59	0.80 to 3.17	0.184
IGHV	2.34	0.99 to 5.52	0.052	-		-
CLL-specific svv						
Age	1.62	0.61 to 4.27	0.330	-		-
Sex	1.58	0.55 to 4.53	0.399	-		-
Rai stage	1.23	0.53 to 2.86	0.624	-		-
LDH	10.32	2.35 to 45.3	0.002	9.27	2.09 to 41.2	0.003
ZAP70	0.91	0.31 to 2.65	0.856	-		-
CD38	1.54	0.52 to 4.57	0.438	-		-
β-2 micro	3.38	1.29 to 8.86	0.013	2.57	0.96 to 6.86	0.060
IGHV	4.00	0.87 to 18.27	0.074	-		-

HR: hazard ratio; CI: confidence interval; PFS: progression-free-survival; TFS: treatment-free-survival; LDH: Lactate DeHydrogenase; OS: overall survival; IGHV: immunoglobulin heavy-chain variable region gene; CLL: chronic lymphocytic leukemia Factors with $p < 0.01$ entered in Multivariate analysis (MV) analysis.
